# Comparison of uninterrupted direct oral anticoagulation with vitamin‐K antagonists during AF‐ablation in the clinical routine. A single center register

**DOI:** 10.1002/clc.23676

**Published:** 2021-07-26

**Authors:** Marian Christoph, Akram Youssef, Jan Svitil, Mathias Forkmann, Yan Huo, Thomas Gaspar, Alexander Francke, Steffen Schoen, Silvio Quick, Carsten Wunderlich

**Affiliations:** ^1^ Technische Universität Dresden, Campus Chemnitz Klinikum Chemnitz Chemnitz Germany; ^2^ Technische Universität Dresden, Heart Center Dresden Technische Universität Dresden Dresden Germany; ^3^ Klinikum Coburg, Department of Cardiology Klinikum Coburg Coburg Germany; ^4^ Technische Universität Dresden Klinikum Pirna Pirna Germany

**Keywords:** AF‐ablation, heparin dosage, oral anticoagulation, periinterventional complications

## Abstract

**Background:**

Uninterrupted direct oral anticoagulation (DOAC) in AF‐ablation is recommended, proven by randomized trials. The outcome and the periinterventional differences between DOACs and VKA in the real world clinical practice are discussed controversial.

**Hypothesis:**

To investigate efficiency and safety of uninterrupted DOAC therapy compared to VKA during AF‐Ablation in real world setting with a focus on periinterventional heparin dosage.

## 1 INTRODUCTION

Thromboembolic events on the one hand and bleeding complications on the other hand represent the most commonly feared complications of AF‐ablation.^1^ The efficacy and safety of continuous periinterventional VKA or DOAC therapy with regard to thromboembolic prophylaxis and bleeding risk has been confirmed in randomized trials.^2^, ^3^, ^4^, ^5^ Based on these studies, the guidelines also support AF‐ablation under ongoing oral anticoagulation either with DOACs or with VKA.^6^ Due to the relatively small number of cases in the randomized trials, further clinical registers are desirable as additional confirmation of the randomized study data in everyday routine. Therefore in the present clinical register the efficiency and safety of uninterrupted DOAC therapy was compared to continued VKA in a real world setting.

In addition, the current study focuses on the different response of heparin to periinterventional ACT in dependence on the ongoing oral anticoagulation.

## 2 METHODS

### 2.1 Study design

The current study was a single‐centre register performed in compliance with the guidelines for good clinical practice and the Declaration of Helsinki. The study was approved by the institutional ethical review board. All data were collected, managed and analyzed at the Heart Centre, University of Dresden (ethics approval: University of Dresden: EK 28409202).

The *primary endpoint* of this study was the occurrence of periinterventional complications during AF ablation procedures (pericardial effusion, transient ischemic attack, stroke, access complications) using two different anticoagulation regimes (VKA or DOACs).

The *secondary endpoints* were the change of hemoglobin levels as marker of periinterventional blood loss, the doses of acquired heparin to reach the target ACT and the mean ACT during the procedure using VKA or DOACs.

### 2.2 Study population and protocol

Eligible subjects were consecutive male or females >18 years of age suffering from symptomatic drug resistant atrial fibrillation (AF) and requiring catheter ablation. No other inclusion criteria were necessary. Exclusion criteria were an interrupted anticoagulation for more than 12 h before the ablation procedure or a bridging of the VKA with heparin. If a patient fulfilled all inclusion criteria and none of the exclusion criteria, the clinical data, intraprocedural data, and complications were analyzed. Five different experienced operators performed all ablation procedures.

The DOAC group consisted of all consecutive patients, which were ablated under uninterrupted anticoagulation with direct oral anticoagulants (DOAC). In these patients the morning dose of the DOAC was given to the patient prior to the procedure. Also the evening dose of apixaban and dabigatran was administered to the patient on the day of the ablation procedure.

The VKA group included all consecutive patients, ablated under continuous administration of vitamin K antagonists (VKA). A statistical based matching of both groups was not performed.

### 2.3 Ablation procedure

If the patients were ≥ 3 weeks on therapeutic OAC no pre‐interventional transesophageal echocardiogram (TOE) was required. The AF ablation procedure was performed under sedation utilizing midazolam fentanyl and propofol. One quadripolar diagnostic catheter was advanced into right ventricular apex and one decapolar catheter into the coronary sinus under fluoroscopic guidance. A single trans‐septal puncture was performed with a steerable intra‐cardiac sheath (Agilis™, St Jude Medical) under fluoroscopy guidance. Upon left atrial access, a bolus of unfractionated Heparin (100 IU/kg) was administered and repeated every 20 min to maintain an activated clotting time between 300 and 350 ms. The correction dose of heparin was determined by the operator. If atrial fibrillation was present during the procedure an electrical cardioversion was performed. An electroanatomic map (EAM) including substrate‐mapping (voltage values higher than 0.5 mV were defined as healthy myocardium) of the left atrium and the pulmonary veins was acquired under fluoroscopic guidance using CARTO or NavX system. During the EAM acquisition selective angiographies of the pulmonary veins were done for fluoroscopic confirmation of catheter position throughout PVI. Subsequently, in all atrial fibrillation procedures a point by point pulmonary vein isolation was performed with pace and ablate technique under guidance of EAM and if necessary under active fluoroscopy (ablation settings: 40 W at an irrigation rate of 15/17 ml/min with a maximum temperature of 43°C, reduction of the RF energy to 30 W at the left atrial posterior wall, intra‐esophageal temperature monitoring with RF stop cut off of 39°C). In case of additional low voltage areas within the left atrium these areas were isolated by point by point creation of additional ablation lines. Finally the conformation of PVI and bidirectional block of all additional lines was performed under EAM and if necessary fluoroscopic guidance. At the end of the procedure, after withdrawal of the intra‐cardiac sheath into the right atrium, in all patients the heparin was antagonized with protamine (with the equal dose of heparin, maximum of 10 000 units). Finally, all sheaths were removed in the EP laboratory, the access sites were compressed manually and a pressure bandage was applied for 6 h.

### 2.4 Follow‐up

Directly after the ablation procedure and 24 h after the ablation a neurological examination was conducted. Additionally, within 24 h after the ablation procedure the access sites were clinically examined. In case of suspect local findings (hematoma, occurrence of new bruits, painful groin) a doppler ultrasound examination of the access site was performed to rule out pseudoaneurysm or AV‐fistula. Further, a transthoracic echocardiography to rule out a pericardial effusion was carried out.

### 2.5 Statistical analysis

Data were tested for normal distribution. Results of continuous variables are expressed as means ± standard deviation. Statistical analyses were done using the 2 tailed, unpaired Student's *t* test. Level of significance was set to *p* < 0.05. Categorical variables are presented as total number with comparison using chi‐square statistics and Fisher exact test. If more than 2 groups were analyzed, a one‐way ANOVA test was performed. Post hoc analyses have been applied using Bonferroni method. Significance level was set to *p* < 0.05.

## 3 RESULTS

### 3.1 Study population, efficacy, and safety endpoints

In total 442 consecutive patients were included in the current analyses. Both treatment groups (VKA *N* = 196 and DOAC *N* = 246) were well balanced in regards to the demographics and clinical baseline characteristics (Table 1). Of note the patients in the VKA group were significantly older (67 ± 8 years) in comparison to the DOAC group (64 ± 9 years). Caused by the higher age in the VKA group the patients in the VKA group had a significantly higher CHA_2_DS_2_‐VASC Score (VKA: 2.7 vs. DOAC: 2.5) and a significantly higher HAS‐BLED‐Score (VKA: 1.3 vs. DOAC: 1.0). The patients in the DOAC group suffered significantly more from diabetes compared to the VKA patients (DOAC: 17.5% vs VKA: 9.7%). There were no relevant differences in gender, co‐morbidities (except diabetes) and concomitant medications.

**TABLE 1:** Baseline characteristics

	Continous VKA	Continous DOAC	*p*‐value
N	196	246	
Male N (%)	111 (56.6)	138 (56.1)	0.92
Age [years] (SD)	67 (8)	64 (9)	**<0.001**
Body hight [meter] (SD)	1.73 (0.09)	1.73 (0.1)	0.53
Body weight [kg] (SD)	87 (16)	87 (17)	0.99
BMI (SD)	28.9 (5)	29.0 (5)	0.77
EHRA Score [mean] (SD)	2.6 (0.6)	2.5 (0.6)	0.42
CHA_2_DS_2_‐VASC Score [mean] (SD)	2.7 (1.3)	2.5 (1.3)	**0.04**
HAS‐BLED Score [mean] (SD)	1.3 (0.8)	1.0 (0.8)	**0.004**
Qualifying risk factors			
Ejection fraction [%] (SD)	55(10)	54 (11)	0.13
Hypertension N (%)	134 (68.4)	189 (76.8)	0.09
Diabetes N (%)	19 (9.7)	43 (17.5)	**0.02**
Coronary heart disease N (%)	9 (4.6)	4 (1.6)	0.09
Renal failure (GFR < 40) N (%)	7 (3.6)	2 (0.8)	0.08

Abbreviation: BMI, body‐mass index (weight in kilograms divided by the square of the height in meters).

### 3.2 Periinterventional complications (primary endpoint)

The periinterventional complications are illustrated in Table 2. In 184/196 VKA patients (93.9%) and in 237/246 DOAC patients (96.3%) the AF‐ablation procedure was performed without any complications. It occurred 3 pericardial effusions (1.5%) in the VKA group, of which 1 of them (0.5%) required cardiac surgery. In the DOAC group there were 3 pericardial effusions (1.2%). None of the pericardial effusion in the DOAC group required surgery. Statistically no significant difference between the two groups could be revealed.

**TABLE 2:** Periinterventional complications (primary endpoint)

Complication [N] (%)	Continous VKA	Continous DOAC	*p*‐value (Bonferroni)
None	184 (93.9)	237 (96.3)	>0.05
Access complications	7 (3.6)	5 (2.0)	>0.05
Pericardial effusion			
No surgery	2 (1.0)	3 (1.2)	>0.05
Require surgery	1 (0.5)	0 (0)	>0.05
TIA	1 (0.5)	1 (0.4)	>0.05
Stroke	1 (0.5)	0 (0)	>0.05

*Note:* Pearson's chi‐squared test, *p* = 0.609.

Abbreviations: access complications, pseudo aneurysm, AV‐fistula; TIA, transient ischemic attack.

All 6 pericardial effusions (3 in the VKA group and 3 in the DOAC group) were treated with a pericardial drainage and re‐transfusion of the drained blood until the pericardial effusion stopped spontaneously or the decision for cardiac surgery was made (1 patient in the VKA group). The use of reversal agents was absolutely avoided to prevent clotting in the pericardial space or in the drainage. Therefore, in case of pericardial tamponades, neither protamine nor fresh frozen plasma, prothrombin complex concentrate or specific reversals for DOACs like idarucizumab and andexanet alfa were used to stop pericardial bleeding.

Due to the re‐transfusion of the drained blood, neither blood concentrates nor platelets or coagulation factors had to be substituted in the further clinical course. However, in the case of pericardial tamponades, VKA or DOACs were postprocedural paused until the patient was clinically stabilized.

In terms of thrombembolic complications there were also no significant differences between the treatment groups (VKA: 1 TIA (0.5%) and 1 stroke (0.5%) vs. DOAC 1 TIA (0.4%) and 0 stroke; *p* > 0.05). The only patient suffered from intraprocedural stroke was on VKA with an INR of 2.0 and in sinus rhythm. In this patient a left facial paresis combined with speech problems, double vision, and vertigo were noticed in the night after ablation. MR tomography revealed a cerebral ischemia in the left thalamus. A conservative treatment was performed.

Further in both groups one TIA was registered. Both patients were in sinus rhythm on admission. One patient suffered from temporary restriction of the visual field. The other patient suffered from temporary weakness of the left arm. In both patients MR tomography could not find any ischaemic lesions.

Further, equal amounts of access site complications (pseudoaneurysm or AV‐fistula) were counted in both treatment groups (VKA 7/196 (3.6%) vs. DOAC 5/246 (2.0%); *p* > 0.05).

### 3.3 Change of periinterventional hemoglobin levels (secondary endpoint)

**FIGURE 1:**
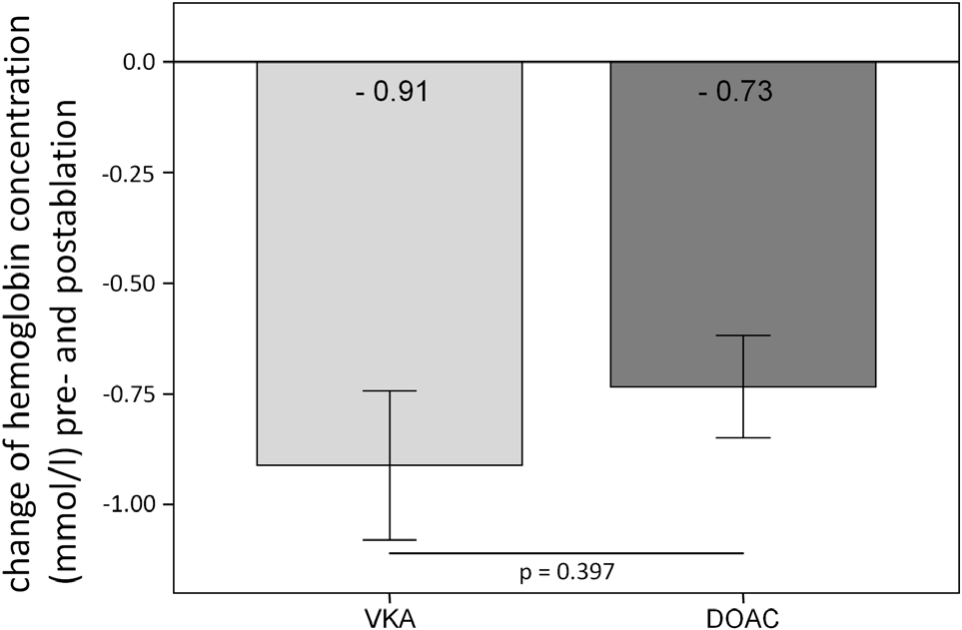
Change of periinterventional hemoglobin levels (secondary endpoint). DOAC, direct oral anticoagulation; VKA, vitamin K antagonist

The comparison of the change of the hemoglobin levels as marker for periinterventional blood loss between both groups is illustrated in Figure 1. In the VKA group the hemoglobin level decreased by 0.91 ± 0.95 mmoL/L and in the DOAC group by 0.73 ± 0.62 mmoL/L during the ablation procedure. There was no statistical difference between both groups (*p* = 0.397).

### 3.4 Mean ACT and heparin doses during AF ablation (secondary endpoint)

**FIGURE 2:**
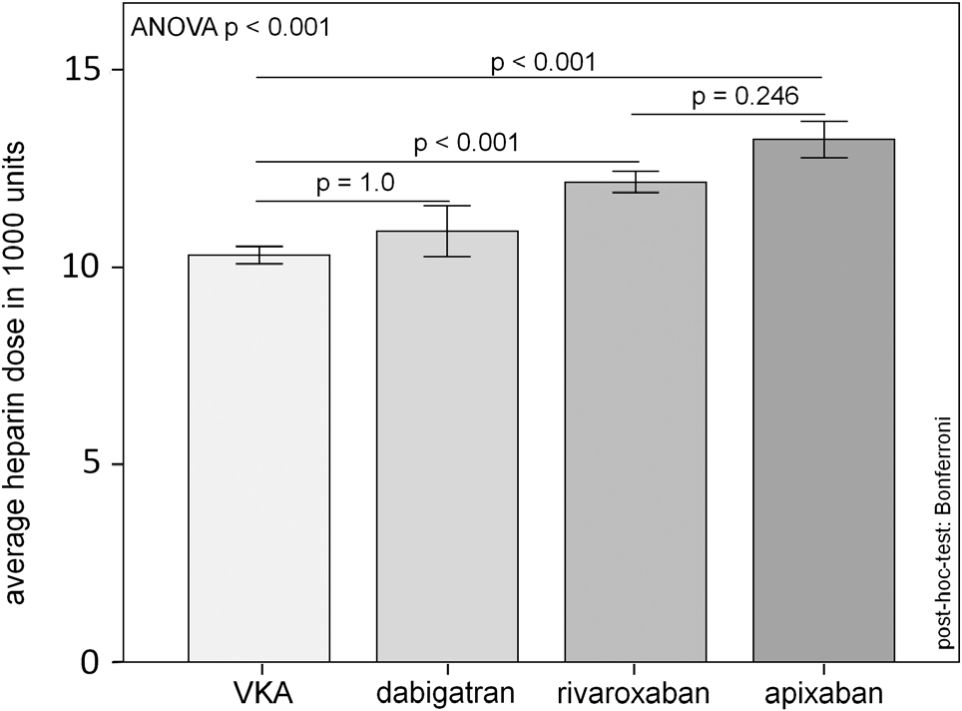
Acquired doses of heparin to reach the target ACT (secondary endpoint)

The comparison of the acquired doses of heparin to reach the target ACT is shown in Figure 2. It could be revealed, that patients treated with direct factor Xa inhibitors apixaban (13 240 ± 3280 units) or rivaroxaban (12 161 ± 3467 units) needed significant higher doses of heparin to reach the target ACT compared to the VKA patients (10 301 ± 3054 units). There was no relevant difference in the heparin dose between the VKA group and the direct thrombin inhibitor dabigatran (10 911 ± 3378 units).

**FIGURE 3:**
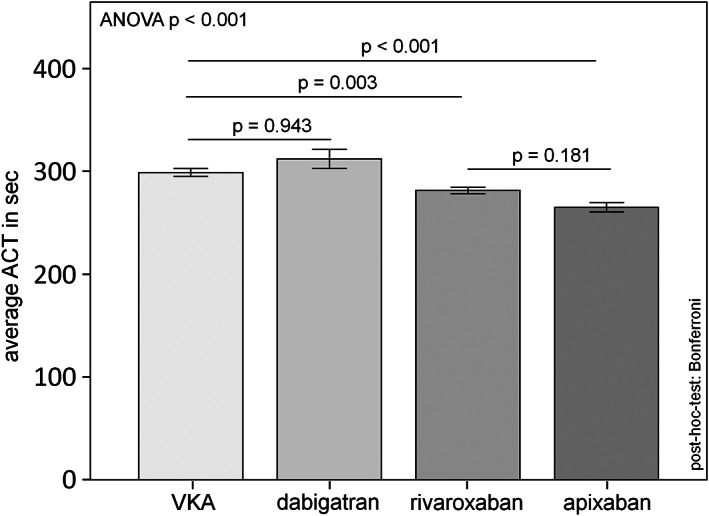
Comparison of mean ACT during AF ablation between VKA and DOAC (secondary endpoint)

Equivalent to these findings the measured ACTs were significantly lower in the Xa inbibitor patients (apixaban: 265 ± 54 s; rivaroxaban: 281 ± 161 s) compared to the VKA and dabigatran group (VKA: 299 ± 54 s; dabigatran: 312 ± 49 s). The respective results are shown in Figure 3. Further, the quotient of the used heparin dose and the resulting ACT was calculated to get a better view of the relative heparin sensitivity in the different anticoagulation groups. Also this heparin/ACT quotient confirmed the significantly higher heparin dose per ACT change in the patients treated with direct Xa inhibitors compared to VKA or dabigatran (results shown in Figure S1).

## 4 DISCUSSION

The findings of the present real world register suggest, that AF‐ablation can be performed very safely even under uninterrupted VKA and DOAC therapy. These results are in line with the conclusions of the previous randomized trials. Thus, this register data are an important scientific supplement to the data from the randomized trials, which could further increase the acceptance of continuous OAC during cardiac interventions. In the current ''every day clinical routine''‐register no preinterventional TOE was required if the patients were ≥ 3 weeks on therapeutic OAC. Further no extended or special access site compressions were used. The access sites were compressed manually and a pressure bandage was applied for 6 h as is also common under paused OAC. Thus this register confirms, that even without special additional precautions, continuous administration of DOACs is safe and effective during AF‐ablation. There were minor differences in baseline characteristics between the current registry and the randomized trials. In the randomized trials, the mean CHA2DS2‐VASc Scores were between 1.5 and 2.4.^3^, ^7^ In contrast, in the current study the mean CHA2DS2‐VASc Score was 2.5 in the DOAC and 2.7 in the VKA group. In light of the aging population with rising co‐morbidities these data support the uninterrupted oral anticoagulation even in patients with higher thromboembolic and bleeding risk. In the current study 1 (0.4%) transient ischemic attack (TIA) in the DOAC group and 1 TIA and 1 stroke (1.0%) were registered in the VKA group. This thrombembolic complication rate was similar to the randomized trials. Thus, in the VENTURE‐AF trial 0.8% of patients and in the AXAFA – AFNET 5 trial 0.3% of patients had thrombembolic events.^5^, ^7^

But in this context it should be noted that the occurrence of intraprocedural strokes and TIAs is not solely depended on pre‐formed thrombi caused by ineffective preinterventional oral anticoagulation, but also on air emboli or ineffective heparinization during the ablation procedure. In the current register all three patients, suffered from stroke or TIA have had a sufficient periprocedural ACT >300 s and air embolization were not noticed during the procedure. Therefore the exact underlying mechanism of the periinterventional thromboembolic events cannot be answered validly in this register.

These low thromboembolism rates were not at the cost of bleeding complications. Puncture site complications are one of the most prevalent clinical fear after groin puncture under uninterrupted anticoagulation with consecutive prolongation of hospital stay. In the current clinical setup in the DOAC group, a puncture site complication occurred in 2.0% of cases. In the VKA group 3.6% of patients suffered from complication at the puncture site. The comparison of these complication rates with other studies is limited, because of the different definitions of puncture site complications. But also in previous trials, complications of the puncture site were very rare. Thus, in a comparable register of Maddox et al, 0.4% of patients had a pseudoaneurysm after AF‐ablation under dabigatran.^8^ But this complication should not be a reason to interrupt anticoagulation in left sided cardiovascular interventions at the cost of a higher thrombembolic risk. Nowadays, there are several strategies to avoid pseudoaneurysms, like sonography guided groin puncture, sufficient manual compression of the puncture site before applying a pressure bandage and the use of closure systems.^9^ Taken together, puncture site complications are more an issue of puncture technique and compression management than of the kind of periinterventional anticoagulation. Pericardial tamponades were also very rare in clinical routine under uninterrupted oral anticoagulation. Thus, in our study we registered 1.2% pericardial tamponades in the DOAC group and 1.5% in the VKA group. This number of pericardial tamponades is completely in line with those of other large AF‐ablation studies irrespective of the anticoagulation strategy.^1^, ^6^, ^8^ This finding suggests, that perioperative caution, rather than the anticoagulation strategy, has the greatest impact on tamponade rates. In the case of pericardial tamponade, complication management is very important for the outcome.^10^ In this context, only one case in the VKA group required cardiac surgery in the current study. All other tamponades could be treated conservatively using pericardial drainage with patient retransfusion of the blood without heparin antagonization to avoid clotting in the pericardial space or in the drainage.

One of the most important findings of this register, that every interventionalist should know, is the different response of heparin to periinterventional ACT in dependence on the ongoing oral anticoagulation. In this context, we observed a significantly increased heparin dose requirement in patients treated with apixaban or rivaroxaban to reach the aim ACT compared to patients treated with VKA or dabigatran. This effect was also observed in the randomized trials.^3^, ^4^, ^5^, ^11^ It is known that ACT‐guided perioperative anticoagulation with heparin helps to prevent thrombembolic complications without the increase of clinical relevant bleeding. Therefore a periinterventional ACT between 300 and 350 s is generally aimed.^12^ Under VKA 100 U/kg should be administered to achieve this ACT value. In contrast, under oral anticoagulation with Xa‐inhibitors the initial dose of heparin should be between 120 and 130 U/kg.^13^ In our register the heparin doses under dabigatran were not significantly different compared to VKA. In our view, this knowledge is very important, because in most clinics during left‐sided procedures, the ACT is often measured discontinuously just every 20 min after the transseptal puncture. But within the first 20 min after the left atrial access the most vulnerable steps of the common procedures like AF‐current application during AF‐ablation, device positioning during LAA closure or clip positioning during interventional mitral valve repair take place. Thus the first bolus application of Heparin should be given in the efficient dosage.

There are some limitations of the current study. First, the study was designed as a register study without randomization of the patients. This could cause a selection bias of the kind of oral anticoagulation of the patients.

A further important limitation is the different distribution of age, CHA2DS2‐VASC, and HAS‐BLED in both groups, which may bias the used statistical tests. In this context, the most important factor is the age of the patients. Thus, it cannot be ruled out with certainty whether the different distribution of the patient's age occurred randomly or whether the age of the patients influence the allocation to the treatment groups (in the current register patients treated with VKA appear to be older than patients treated with DOACs). This higher age in the VKA group triggers the higher CHA2DS2‐VASC score. Further, the patients in the VKA group have higher HAS‐BLED scores because the point ''labile INR'' is not applicable for DOAC patients.

Second, like the study populations in the randomized trials, the study population in this register was relatively small with very low event rates. Consequently, the abovementioned findings have to be confirmed by larger outcome studies.

## 5 CONCLUSIONS

The use of uninterrupted DOACs during AF‐ablation is safe and the amount of thrombembolic and bleeding events was very low and similar to that for continuing VKA in the clinical routine.

Of note, under the factor Xa inhibitors, there is a significant lower anticoagulatory effect of heparin, as measured by the activated clotting time, requiring higher periinterventional heparin doses. These findings should be translated to other left‐sited cardiovascular interventions.

## CONFLICT OF INTEREST

The authors declare no potential conflict of interest.

## Supporting information

**Figure S1** Comparison ACT/heparin quotient between VKA and DOAC (secondary endpoint)

## Data Availability

The data that support the findings of this study are available from the corresponding author, MC, upon reasonable request.
