# Supplementary figures and images for: Comparison of uninterrupted direct oral anticoagulation with vitamin‐K antagonists during AF‐ablation in the clinical routine. A single center register
Source: Clin Cardiol. 2021 Jul 26;44(9):1243–8. doi: 10.1002/clc.23676 (PMC8428011; doi:10.1002/clc.23676)

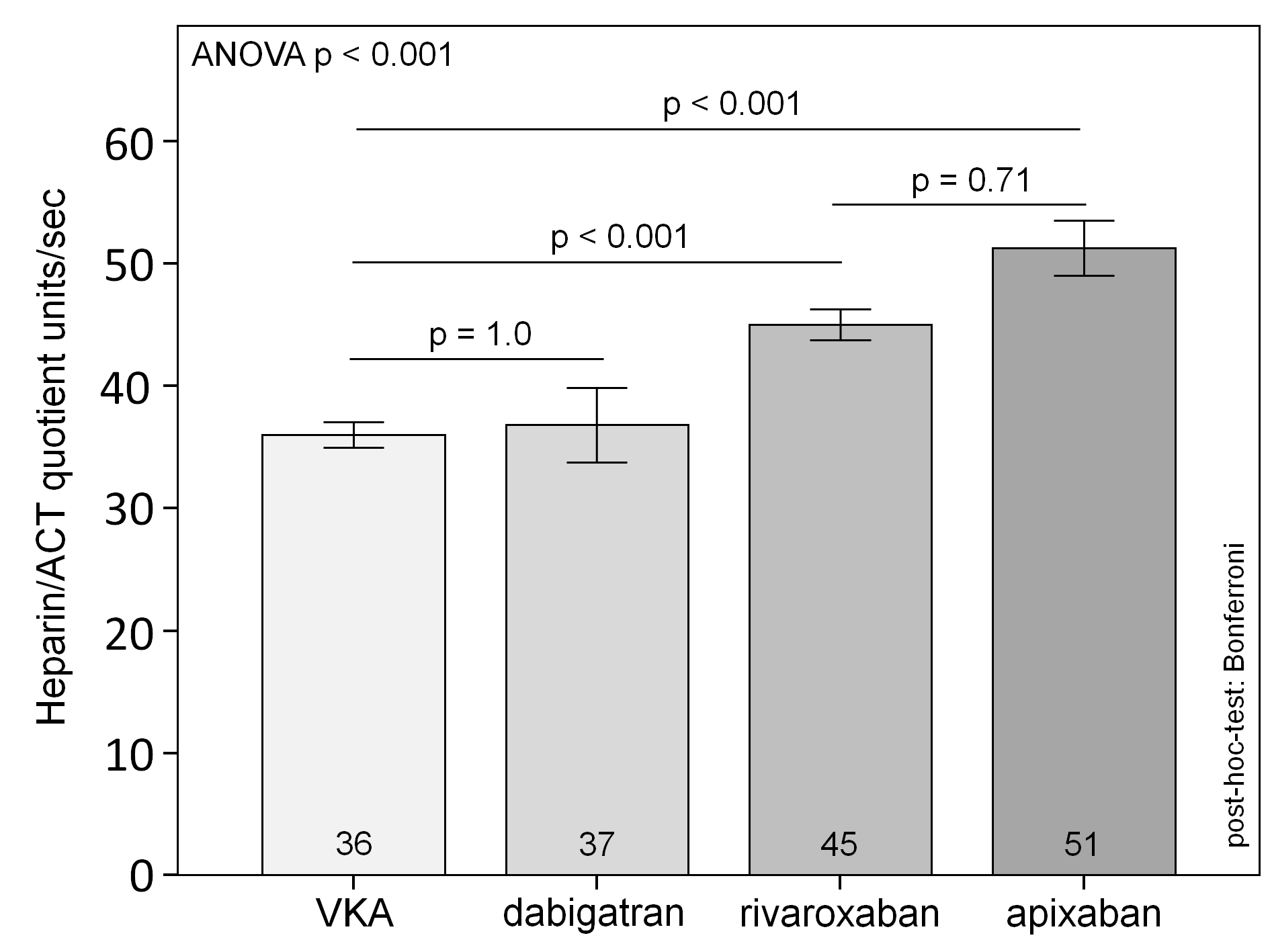

Supplement: Supplementary file 1 — Figure S1 Comparison ACT/heparin quotient between VKA and DOAC (secondary endpoint) [file CLC-44-1243-s001.tif]
